# 2α-Acet­oxy-5α-methoxy­caryophyll-8(15)-en-3-one

**DOI:** 10.1107/S1600536809018856

**Published:** 2009-05-23

**Authors:** Wen Zhang, Hong-Quan Duan

**Affiliations:** a School of Pharmacy, Tianjin Medical University, Tianjin 300070, People’s Republic of China

## Abstract

The title compound, C_18_H_28_O_4_, crystallizes with two mol­ecules in the asymmetric unit. Both mol­ecules have similar conformations of nine-membered rings, which are *trans*-fused with cyclo­butane fragments. The puckering amplitudes (*q*2) of the cyclo­butane rings are 0.2451 (2) and 0.2526 (2) Å.

## Related literature

For the biological activity of the title compound, see: Houghton (1984 ▶); Yamamoto *et al.* (1993 ▶); Yoshida *et al.* (1978 ▶). For puckering amplitude, see: Cremer & Pople (1975 ▶). For a related structure of the carryophyllane type, see: Collado *et al.* (1997 ▶).
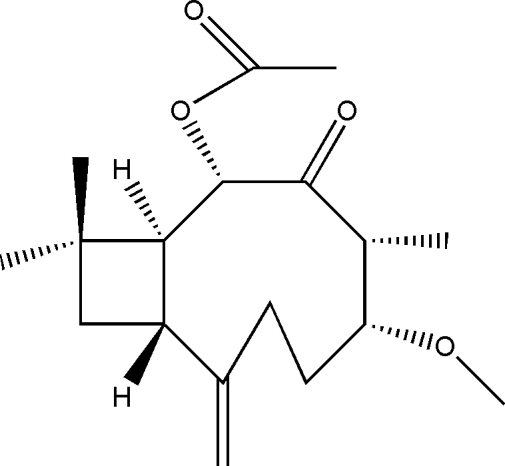


## Experimental

### 

#### Crystal data

C_18_H_28_O_4_
*M* *_r_* = 308.40
Orthorhombic, 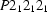

*a* = 9.2924 (19) Å
*b* = 17.858 (4) Å
*c* = 21.451 (4) Å
*V* = 3559.6 (12) Å^3^
*Z* = 8
Mo *K*α radiation
μ = 0.08 mm^−1^
*T* = 113 K
0.18 × 0.16 × 0.08 mm

#### Data collection

Rigaku Saturn diffractometer
Absorption correction: multi-scan (*CrystalClear*; Rigaku, 2005 ▶) *T*_min_ = 0.986, *T*_max_ = 0.994
21386 measured reflections
3534 independent reflections
3031 reflections with *I* > 2σ(*I*)
*R*_int_ = 0.066

#### Refinement

*R*[*F* ^2^ > 2σ(*F* ^2^)] = 0.049
*wR*(*F* ^2^) = 0.123
*S* = 1.08
3534 reflections
408 parameters
H-atom parameters constrained
Δρ_max_ = 0.19 e Å^−3^
Δρ_min_ = −0.20 e Å^−3^

### 

Data collection: *CrystalClear* (Rigaku, 2005 ▶); cell refinement: *CrystalClear*; data reduction: *CrystalClear*; program(s) used to solve structure: *SHELXTL* (Sheldrick, 2008 ▶); program(s) used to refine structure: *SHELXTL*; molecular graphics: *SHELXTL*; software used to prepare material for publication: *SHELXTL*.

## Supplementary Material

Crystal structure: contains datablocks I, global. DOI: 10.1107/S1600536809018856/ya2089sup1.cif

Structure factors: contains datablocks I. DOI: 10.1107/S1600536809018856/ya2089Isup2.hkl

Additional supplementary materials:  crystallographic information; 3D view; checkCIF report

## Figures and Tables

**Fig. 1.**
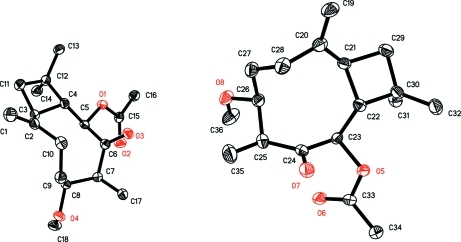
Molecular structure of the title compound (I). Displacement ellipsoids are drawn at the 35% probability level; H atoms are omitted for clarity.

## supplementary crystallographic information

### Comment

The genus Buddleja has widespread use in folk medicine (Houghton, 1984). *B. davidii* have been reported to have bactericidal and cannabimimetic antiinflammatory properties (Yamamoto *et al.*, 1993; Yoshida *et al.*, 1978). The present paper reports the structure of the title compound, a caryophyllane-type sesquiterpene, isolated from the bark of *Buddleja davidii* Franch.

The asymmetric unit contains two chemically identical and conformationally similar molecules (Fig. 1). The 9-membered ring is *trans*-fused with cyclobutane; the Cremer & Pople puckering amplitudes (q2) of the cyclobutane rings are 0.2451 (2) and 0.2526 (2)Å (Cremer & Pople, 1975).

### Experimental

Air-dried roots (2 kg) were extracted with 95% EtOH. The extract was suspended in water and extracted with petroleum ether, EtOAc and n-BuOH, respectively. The EtOAc extract was evaporated to dryness under reduced pressure. The residue (48.2 g) was chromatographed on a silica gel column with a gradient of petroleum ether–EtOAc –MeOH (3: 1: 0→0: 0: 1, *v*/*v*) to afford 34 fractions (F_1_– F_34_), pooled by common TLC characteristics. F_5_ (4.2 g) was separated by a Toyopearl HW-40 column (CH_2_Cl_2_–MeOH, 1: 1, *v*/*v*) to afford 7 fractions (Fractions a-g). Fraction b (0.4 g) was separated by preparative HPLC with MeOH–H_2_O (7: 3, *v*/*v*) to give 17 fractions (F_1_– F_17_). Fraction 14 (0.1 g) was separated by preparative TLC with CH_2_Cl_2_–MeOH, (9: 1, *v*/*v*) to give 2α-acetoxy-5α-methoxy-*enantio*-caryophylla-8(15)-en-3-one (20.0 mg, 3.0 ml/min, t_R_ = 36.3 min) as colourless plate-shaped crystals. The structure of compound is consistent with its IR, MS, 1*H*-NMR, ^13^C-NMR and two-dimensional-NMR spectra.

### Refinement

With no anomalous scatterers with *Z*Si present in the molecule and the use of Mo irraditaion, all Friedel equivalents were merged. The absolute configuration of the molecule was assigned on the basis of the known configuration of carryophyllane-type sesquiterpene skeleton, *e.g.* see Collado *et al.*, 1997.

All H atoms were placed in calculated positions, with C—H = 0.93–0.98 Å, and included in the final cycles of refinement in a riding model approximation, with *U*_iso_(H) = 1.2 times *U*_eq_(C) (1.5 *U*_eq_(C) for methyl H atoms).

### Crystal data

**Table:**

C_18_H_28_O_4_	*F*(000) = 1344
*M**_r_* = 308.40	*D*_x_ = 1.151 Mg m^−^^3^
Orthorhombic, *P*2_1_2_1_2_1_	Mo *K*α radiation, λ = 0.71073 Å
Hall symbol: P 2ac 2ab	Cell parameters from 7216 reflections
*a* = 9.2924 (19) Å	θ = 1.9–27.8°
*b* = 17.858 (4) Å	µ = 0.08 mm^−^^1^
*c* = 21.451 (4) Å	*T* = 113 K
*V* = 3559.6 (12) Å^3^	Plate, colourless
*Z* = 8	0.18 × 0.16 × 0.08 mm

### Data collection

**Table:**

Rigaku Saturn diffractometer	3534 independent reflections
Radiation source: rotating anode	3031 reflections with *I* > 2σ(*I*)
confocal	*R*_int_ = 0.066
ω scans	θ_max_ = 25.0°, θ_min_ = 1.9°
Absorption correction: multi-scan (*CrystalClear*; Rigaku, 2005)	*h* = −11→7
*T*_min_ = 0.986, *T*_max_ = 0.994	*k* = −21→18
21386 measured reflections	*l* = −25→25

### Refinement

**Table:**

Refinement on *F*^2^	Secondary atom site location: difference Fourier map
Least-squares matrix: full	Hydrogen site location: inferred from neighbouring sites
*R*[*F*^2^ > 2σ(*F*^2^)] = 0.049	H-atom parameters constrained
*wR*(*F*^2^) = 0.123	*w* = 1/[σ^2^(*F*_o_^2^) + (0.0685*P*)^2^ + 0.2388*P*] where *P* = (*F*_o_^2^ + 2*F*_c_^2^)/3
*S* = 1.08	(Δ/σ)_max_ < 0.001
3534 reflections	Δρ_max_ = 0.19 e Å^−^^3^
408 parameters	Δρ_min_ = −0.20 e Å^−^^3^
0 restraints	Extinction correction: *SHELXTL* (Sheldrick, 2008), Fc^*^=kFc[1+0.001xFc^2^λ^3^/sin(2θ)]^-1/4^
Primary atom site location: structure-invariant direct methods	Extinction coefficient: 0.0185 (15)

### Special details

**Table:**

Geometry. All e.s.d.'s (except the e.s.d. in the dihedral angle between two l.s. planes) are estimated using the full covariance matrix. The cell e.s.d.'s are taken into account individually in the estimation of e.s.d.'s in distances, angles and torsion angles; correlations between e.s.d.'s in cell parameters are only used when they are defined by crystal symmetry. An approximate (isotropic) treatment of cell e.s.d.'s is used for estimating e.s.d.'s involving l.s. planes.
Refinement. Refinement of *F*^2^ against ALL reflections. The weighted *R*-factor *wR* and goodness of fit *S* are based on *F*^2^, conventional *R*-factors *R* are based on *F*, with *F* set to zero for negative *F*^2^. The threshold expression of *F*^2^ > σ(*F*^2^) is used only for calculating *R*-factors(gt) *etc*. and is not relevant to the choice of reflections for refinement. *R*-factors based on *F*^2^ are statistically about twice as large as those based on *F*, and *R*- factors based on ALL data will be even larger.

### Fractional atomic coordinates and isotropic or equivalent isotropic displacement parameters (Å^2^)

**Table:**

	*x*	*y*	*z*	*U*_iso_*/*U*_eq_	
O1	0.5582 (3)	0.12567 (11)	0.81709 (10)	0.0301 (5)	
O2	0.7395 (3)	0.20496 (12)	0.83806 (13)	0.0437 (7)	
O3	0.6143 (3)	0.17341 (12)	0.69939 (10)	0.0344 (6)	
O4	0.4326 (3)	0.42123 (11)	0.71852 (11)	0.0363 (6)	
O5	0.8704 (3)	0.08791 (11)	0.03030 (10)	0.0305 (5)	
O6	0.8493 (3)	0.16830 (12)	0.11031 (12)	0.0406 (6)	
O7	0.5906 (3)	0.12357 (12)	0.00668 (11)	0.0407 (6)	
O8	0.4325 (3)	0.03567 (13)	0.20611 (11)	0.0408 (6)	
C1	0.0120 (5)	0.2194 (2)	0.6862 (2)	0.0520 (11)	
H1A	−0.0571	0.2055	0.7154	0.062*	
H1B	−0.0153	0.2311	0.6457	0.062*	
C2	0.1489 (4)	0.22230 (19)	0.70240 (18)	0.0372 (9)	
C3	0.1944 (4)	0.20341 (18)	0.76783 (17)	0.0326 (8)	
H3	0.2034	0.2496	0.7922	0.039*	
C4	0.3297 (4)	0.15383 (17)	0.77841 (15)	0.0301 (7)	
H4	0.3386	0.1188	0.7434	0.036*	
C5	0.4763 (4)	0.18797 (15)	0.79133 (15)	0.0269 (7)	
H5	0.4674	0.2278	0.8225	0.032*	
C6	0.5546 (4)	0.21756 (17)	0.73375 (15)	0.0288 (7)	
C7	0.5603 (4)	0.30145 (16)	0.72253 (16)	0.0316 (8)	
H7	0.6109	0.3225	0.7586	0.038*	
C8	0.4135 (4)	0.34110 (16)	0.72065 (16)	0.0314 (8)	
H8	0.3590	0.3280	0.7582	0.038*	
C9	0.3222 (4)	0.32443 (19)	0.66349 (18)	0.0387 (9)	
H9A	0.3793	0.3358	0.6269	0.046*	
H9B	0.2412	0.3587	0.6637	0.046*	
C10	0.2631 (4)	0.24503 (19)	0.65604 (16)	0.0369 (8)	
H10A	0.2234	0.2402	0.6144	0.044*	
H10B	0.3427	0.2101	0.6593	0.044*	
C11	0.1069 (4)	0.1455 (2)	0.80510 (18)	0.0415 (9)	
H11A	0.0552	0.1101	0.7791	0.050*	
H11B	0.0434	0.1676	0.8359	0.050*	
C12	0.2483 (4)	0.11359 (18)	0.83302 (16)	0.0338 (8)	
C13	0.2632 (4)	0.02850 (17)	0.83394 (17)	0.0371 (8)	
H13A	0.1978	0.0079	0.8640	0.056*	
H13B	0.3600	0.0153	0.8450	0.056*	
H13C	0.2412	0.0088	0.7934	0.056*	
C14	0.2787 (5)	0.1458 (2)	0.89726 (17)	0.0424 (9)	
H14A	0.3761	0.1347	0.9089	0.064*	
H14B	0.2140	0.1239	0.9270	0.064*	
H14C	0.2651	0.1990	0.8964	0.064*	
C15	0.6904 (4)	0.14231 (19)	0.83953 (16)	0.0325 (8)	
C16	0.7598 (4)	0.07496 (18)	0.86604 (17)	0.0380 (8)	
H16A	0.7709	0.0811	0.9102	0.057*	
H16B	0.8527	0.0682	0.8472	0.057*	
H16C	0.7010	0.0319	0.8579	0.057*	
C17	0.6540 (4)	0.31971 (19)	0.6662 (2)	0.0456 (10)	
H17A	0.6494	0.3725	0.6579	0.068*	
H17B	0.6198	0.2925	0.6306	0.068*	
H17C	0.7518	0.3057	0.6748	0.068*	
C18	0.4817 (5)	0.45335 (18)	0.77483 (17)	0.0456 (10)	
H18A	0.5807	0.4400	0.7814	0.068*	
H18B	0.4246	0.4350	0.8089	0.068*	
H18C	0.4732	0.5069	0.7725	0.068*	
C19	0.4679 (5)	−0.1737 (2)	0.00807 (18)	0.0519 (11)	
H19A	0.3720	−0.1800	−0.0032	0.062*	
H19B	0.5337	−0.2121	0.0014	0.062*	
C20	0.5109 (4)	−0.10977 (19)	0.03360 (16)	0.0382 (9)	
C21	0.6660 (4)	−0.09676 (17)	0.05160 (15)	0.0317 (8)	
H21	0.6735	−0.0954	0.0972	0.038*	
C22	0.7481 (4)	−0.02867 (16)	0.02388 (14)	0.0286 (7)	
H22	0.7126	−0.0188	−0.0183	0.034*	
C23	0.7560 (4)	0.04451 (16)	0.05969 (15)	0.0264 (7)	
H23	0.7796	0.0348	0.1035	0.032*	
C24	0.6160 (4)	0.09050 (17)	0.05509 (16)	0.0300 (8)	
C25	0.5209 (4)	0.09690 (19)	0.11215 (17)	0.0388 (9)	
H25	0.5758	0.1268	0.1422	0.047*	
C26	0.4905 (4)	0.02218 (17)	0.14503 (15)	0.0331 (8)	
H26	0.5805	−0.0060	0.1489	0.040*	
C27	0.3779 (4)	−0.0270 (2)	0.11343 (17)	0.0411 (9)	
H27A	0.2858	−0.0016	0.1162	0.049*	
H27B	0.3699	−0.0731	0.1370	0.049*	
C28	0.4044 (4)	−0.0475 (2)	0.04438 (16)	0.0410 (9)	
H28A	0.3133	−0.0618	0.0258	0.049*	
H28B	0.4382	−0.0032	0.0227	0.049*	
C29	0.7857 (4)	−0.14730 (18)	0.02549 (18)	0.0407 (9)	
H29A	0.7620	−0.1700	−0.0143	0.049*	
H29B	0.8193	−0.1847	0.0549	0.049*	
C30	0.8868 (4)	−0.07856 (17)	0.01989 (16)	0.0338 (8)	
C31	0.9849 (4)	−0.0714 (2)	0.07619 (16)	0.0391 (9)	
H31A	1.0303	−0.0232	0.0759	0.059*	
H31B	1.0572	−0.1098	0.0747	0.059*	
H31C	0.9292	−0.0769	0.1136	0.059*	
C32	0.9716 (5)	−0.0720 (2)	−0.04019 (17)	0.0429 (10)	
H32A	1.0432	−0.1107	−0.0417	0.064*	
H32B	1.0177	−0.0239	−0.0419	0.064*	
H32C	0.9076	−0.0772	−0.0751	0.064*	
C33	0.9055 (4)	0.15150 (17)	0.06118 (16)	0.0333 (8)	
C34	1.0164 (5)	0.19589 (19)	0.0281 (2)	0.0503 (11)	
H34A	0.9948	0.2482	0.0321	0.075*	
H34B	1.0171	0.1823	−0.0152	0.075*	
H34C	1.1091	0.1859	0.0460	0.075*	
C35	0.3847 (5)	0.1418 (2)	0.0989 (2)	0.0607 (12)	
H35A	0.3342	0.1197	0.0644	0.091*	
H35B	0.4102	0.1924	0.0887	0.091*	
H35C	0.3241	0.1415	0.1351	0.091*	
C36	0.5340 (5)	0.0672 (2)	0.24869 (17)	0.0557 (12)	
H36A	0.5556	0.1176	0.2365	0.084*	
H36B	0.6207	0.0379	0.2482	0.084*	
H36C	0.4940	0.0671	0.2899	0.084*	

### Atomic displacement parameters (Å^2^)

**Table:**

	*U*^11^	*U*^22^	*U*^33^	*U*^12^	*U*^13^	*U*^23^
O1	0.0312 (13)	0.0250 (11)	0.0341 (12)	−0.0012 (10)	−0.0041 (10)	0.0012 (9)
O2	0.0355 (15)	0.0323 (13)	0.0633 (17)	−0.0060 (13)	−0.0092 (14)	0.0009 (12)
O3	0.0398 (15)	0.0303 (11)	0.0331 (12)	0.0031 (11)	0.0057 (11)	−0.0027 (10)
O4	0.0465 (16)	0.0248 (11)	0.0375 (13)	0.0036 (11)	0.0041 (12)	−0.0005 (10)
O5	0.0358 (14)	0.0260 (11)	0.0298 (12)	−0.0039 (11)	0.0045 (11)	−0.0010 (9)
O6	0.0532 (17)	0.0298 (12)	0.0389 (14)	−0.0106 (13)	0.0117 (13)	−0.0071 (10)
O7	0.0459 (16)	0.0385 (13)	0.0376 (14)	0.0101 (12)	0.0036 (12)	0.0097 (11)
O8	0.0409 (15)	0.0476 (14)	0.0340 (13)	−0.0134 (13)	0.0081 (12)	−0.0094 (11)
C1	0.040 (2)	0.052 (2)	0.065 (3)	0.003 (2)	−0.013 (2)	0.005 (2)
C2	0.030 (2)	0.0335 (18)	0.048 (2)	0.0053 (16)	−0.0019 (18)	−0.0046 (16)
C3	0.0274 (19)	0.0268 (16)	0.044 (2)	0.0030 (14)	0.0025 (16)	−0.0029 (14)
C4	0.0279 (18)	0.0309 (16)	0.0317 (17)	0.0019 (15)	0.0027 (15)	−0.0044 (14)
C5	0.0294 (18)	0.0210 (15)	0.0302 (16)	0.0021 (14)	−0.0006 (15)	−0.0007 (12)
C6	0.0263 (18)	0.0302 (16)	0.0300 (17)	0.0004 (15)	−0.0038 (15)	−0.0006 (14)
C7	0.0302 (19)	0.0271 (16)	0.0374 (19)	−0.0007 (15)	0.0027 (15)	0.0018 (14)
C8	0.034 (2)	0.0235 (16)	0.0365 (18)	0.0009 (15)	0.0045 (16)	−0.0028 (14)
C9	0.038 (2)	0.0407 (19)	0.0371 (19)	0.0023 (18)	−0.0005 (17)	0.0021 (16)
C10	0.036 (2)	0.0413 (19)	0.0331 (17)	0.0040 (18)	−0.0087 (17)	−0.0056 (15)
C11	0.032 (2)	0.0402 (19)	0.052 (2)	−0.0053 (17)	0.0044 (18)	−0.0064 (17)
C12	0.0300 (19)	0.0341 (17)	0.0374 (19)	−0.0022 (16)	0.0041 (17)	−0.0033 (14)
C13	0.036 (2)	0.0348 (17)	0.040 (2)	−0.0078 (17)	0.0043 (18)	−0.0029 (15)
C14	0.046 (2)	0.0402 (19)	0.041 (2)	−0.0071 (18)	0.0114 (18)	−0.0039 (16)
C15	0.0314 (19)	0.0374 (19)	0.0286 (16)	0.0009 (16)	0.0033 (15)	0.0006 (15)
C16	0.036 (2)	0.0395 (19)	0.0381 (19)	0.0001 (18)	−0.0002 (18)	0.0076 (15)
C17	0.041 (2)	0.0347 (19)	0.061 (3)	0.0099 (19)	0.018 (2)	0.0110 (18)
C18	0.069 (3)	0.0293 (17)	0.039 (2)	−0.0051 (19)	0.015 (2)	−0.0036 (15)
C19	0.056 (3)	0.051 (2)	0.048 (2)	−0.026 (2)	0.008 (2)	−0.0148 (18)
C20	0.044 (2)	0.0401 (19)	0.0303 (18)	−0.0108 (18)	0.0044 (17)	−0.0048 (14)
C21	0.043 (2)	0.0243 (16)	0.0275 (17)	−0.0042 (16)	0.0050 (16)	−0.0017 (13)
C22	0.0347 (19)	0.0265 (15)	0.0245 (16)	0.0016 (16)	−0.0016 (16)	−0.0009 (13)
C23	0.0259 (18)	0.0272 (15)	0.0261 (16)	−0.0035 (15)	0.0042 (14)	0.0011 (12)
C24	0.034 (2)	0.0220 (15)	0.0337 (18)	0.0007 (15)	0.0018 (16)	−0.0006 (14)
C25	0.040 (2)	0.0369 (18)	0.0399 (19)	0.0042 (17)	0.0093 (18)	−0.0034 (15)
C26	0.034 (2)	0.0336 (16)	0.0320 (17)	−0.0040 (17)	0.0044 (16)	−0.0056 (13)
C27	0.034 (2)	0.047 (2)	0.043 (2)	−0.0084 (18)	0.0052 (18)	−0.0137 (17)
C28	0.035 (2)	0.052 (2)	0.036 (2)	−0.0084 (19)	−0.0053 (17)	−0.0073 (16)
C29	0.055 (3)	0.0266 (16)	0.040 (2)	0.0016 (17)	0.0062 (19)	0.0008 (15)
C30	0.042 (2)	0.0277 (17)	0.0315 (18)	0.0037 (16)	0.0026 (17)	−0.0026 (14)
C31	0.038 (2)	0.041 (2)	0.038 (2)	0.0105 (18)	−0.0021 (17)	0.0048 (15)
C32	0.052 (3)	0.039 (2)	0.037 (2)	0.0101 (19)	0.0075 (19)	0.0000 (15)
C33	0.038 (2)	0.0227 (16)	0.0398 (19)	−0.0014 (15)	0.0030 (17)	0.0014 (15)
C34	0.050 (3)	0.0349 (19)	0.066 (3)	−0.0073 (19)	0.022 (2)	−0.0037 (17)
C35	0.058 (3)	0.055 (3)	0.069 (3)	0.020 (2)	0.018 (3)	0.007 (2)
C36	0.060 (3)	0.074 (3)	0.033 (2)	−0.030 (2)	0.007 (2)	−0.0087 (18)

### Geometric parameters (Å, °)

**Table:**

O1—C15	1.353 (4)	C17—H17A	0.9600
O1—C5	1.456 (4)	C17—H17B	0.9600
O2—C15	1.209 (4)	C17—H17C	0.9600
O3—C6	1.214 (4)	C18—H18A	0.9600
O4—C18	1.413 (4)	C18—H18B	0.9600
O4—C8	1.443 (4)	C18—H18C	0.9600
O5—C33	1.355 (4)	C19—C20	1.328 (5)
O5—C23	1.459 (4)	C19—H19A	0.9300
O6—C33	1.214 (4)	C19—H19B	0.9300
O7—C24	1.218 (4)	C20—C28	1.507 (5)
O8—C36	1.428 (5)	C20—C21	1.510 (5)
O8—C26	1.437 (4)	C21—C29	1.538 (5)
C1—C2	1.319 (6)	C21—C22	1.554 (4)
C1—H1A	0.9300	C21—H21	0.9800
C1—H1B	0.9300	C22—C23	1.517 (4)
C2—C3	1.504 (5)	C22—C30	1.569 (5)
C2—C10	1.510 (5)	C22—H22	0.9800
C3—C11	1.540 (5)	C23—C24	1.542 (5)
C3—C4	1.554 (5)	C23—H23	0.9800
C3—H3	0.9800	C24—C25	1.514 (5)
C4—C5	1.518 (5)	C25—C35	1.525 (6)
C4—C12	1.569 (5)	C25—C26	1.535 (5)
C4—H4	0.9800	C25—H25	0.9800
C5—C6	1.528 (4)	C26—C27	1.525 (5)
C5—H5	0.9800	C26—H26	0.9800
C6—C7	1.518 (4)	C27—C28	1.546 (5)
C7—C17	1.524 (5)	C27—H27A	0.9700
C7—C8	1.537 (5)	C27—H27B	0.9700
C7—H7	0.9800	C28—H28A	0.9700
C8—C9	1.521 (5)	C28—H28B	0.9700
C8—H8	0.9800	C29—C30	1.550 (5)
C9—C10	1.529 (5)	C29—H29A	0.9700
C9—H9A	0.9700	C29—H29B	0.9700
C9—H9B	0.9700	C30—C32	1.515 (5)
C10—H10A	0.9700	C30—C31	1.518 (5)
C10—H10B	0.9700	C31—H31A	0.9600
C11—C12	1.553 (5)	C31—H31B	0.9600
C11—H11A	0.9700	C31—H31C	0.9600
C11—H11B	0.9700	C32—H32A	0.9600
C12—C14	1.519 (5)	C32—H32B	0.9600
C12—C13	1.526 (5)	C32—H32C	0.9600
C13—H13A	0.9600	C33—C34	1.481 (5)
C13—H13B	0.9600	C34—H34A	0.9600
C13—H13C	0.9600	C34—H34B	0.9600
C14—H14A	0.9600	C34—H34C	0.9600
C14—H14B	0.9600	C35—H35A	0.9600
C14—H14C	0.9600	C35—H35B	0.9600
C15—C16	1.478 (5)	C35—H35C	0.9600
C16—H16A	0.9600	C36—H36A	0.9600
C16—H16B	0.9600	C36—H36B	0.9600
C16—H16C	0.9600	C36—H36C	0.9600
			
C15—O1—C5	116.2 (2)	H18A—C18—H18C	109.5
C18—O4—C8	114.5 (2)	H18B—C18—H18C	109.5
C33—O5—C23	114.2 (2)	C20—C19—H19A	120.0
C36—O8—C26	113.7 (3)	C20—C19—H19B	120.0
C2—C1—H1A	120.0	H19A—C19—H19B	120.0
C2—C1—H1B	120.0	C19—C20—C28	120.0 (4)
H1A—C1—H1B	120.0	C19—C20—C21	121.7 (4)
C1—C2—C3	120.5 (4)	C28—C20—C21	118.3 (3)
C1—C2—C10	121.0 (4)	C20—C21—C29	120.4 (3)
C3—C2—C10	118.5 (3)	C20—C21—C22	119.4 (3)
C2—C3—C11	119.1 (3)	C29—C21—C22	88.0 (2)
C2—C3—C4	119.4 (3)	C20—C21—H21	109.1
C11—C3—C4	88.2 (3)	C29—C21—H21	109.1
C2—C3—H3	109.4	C22—C21—H21	109.1
C11—C3—H3	109.4	C23—C22—C21	120.3 (3)
C4—C3—H3	109.4	C23—C22—C30	118.5 (3)
C5—C4—C3	121.6 (3)	C21—C22—C30	88.8 (2)
C5—C4—C12	118.7 (3)	C23—C22—H22	109.2
C3—C4—C12	88.9 (3)	C21—C22—H22	109.2
C5—C4—H4	108.7	C30—C22—H22	109.2
C3—C4—H4	108.7	O5—C23—C22	105.9 (2)
C12—C4—H4	108.7	O5—C23—C24	107.7 (2)
O1—C5—C4	103.4 (2)	C22—C23—C24	112.7 (3)
O1—C5—C6	108.8 (3)	O5—C23—H23	110.1
C4—C5—C6	114.7 (3)	C22—C23—H23	110.1
O1—C5—H5	109.9	C24—C23—H23	110.1
C4—C5—H5	109.9	O7—C24—C25	122.6 (3)
C6—C5—H5	109.9	O7—C24—C23	118.5 (3)
O3—C6—C7	121.9 (3)	C25—C24—C23	118.8 (3)
O3—C6—C5	118.9 (3)	C24—C25—C35	111.9 (3)
C7—C6—C5	119.1 (3)	C24—C25—C26	114.4 (3)
C6—C7—C17	110.9 (3)	C35—C25—C26	112.9 (3)
C6—C7—C8	115.3 (3)	C24—C25—H25	105.6
C17—C7—C8	112.8 (3)	C35—C25—H25	105.6
C6—C7—H7	105.6	C26—C25—H25	105.6
C17—C7—H7	105.6	O8—C26—C27	104.2 (3)
C8—C7—H7	105.6	O8—C26—C25	110.0 (3)
O4—C8—C9	103.7 (3)	C27—C26—C25	115.0 (3)
O4—C8—C7	110.4 (3)	O8—C26—H26	109.2
C9—C8—C7	115.2 (3)	C27—C26—H26	109.2
O4—C8—H8	109.1	C25—C26—H26	109.2
C9—C8—H8	109.1	C26—C27—C28	116.9 (3)
C7—C8—H8	109.1	C26—C27—H27A	108.1
C8—C9—C10	117.8 (3)	C28—C27—H27A	108.1
C8—C9—H9A	107.9	C26—C27—H27B	108.1
C10—C9—H9A	107.9	C28—C27—H27B	108.1
C8—C9—H9B	107.9	H27A—C27—H27B	107.3
C10—C9—H9B	107.9	C20—C28—C27	115.3 (3)
H9A—C9—H9B	107.2	C20—C28—H28A	108.5
C2—C10—C9	115.6 (3)	C27—C28—H28A	108.5
C2—C10—H10A	108.4	C20—C28—H28B	108.5
C9—C10—H10A	108.4	C27—C28—H28B	108.5
C2—C10—H10B	108.4	H28A—C28—H28B	107.5
C9—C10—H10B	108.4	C21—C29—C30	90.1 (2)
H10A—C10—H10B	107.4	C21—C29—H29A	113.6
C3—C11—C12	90.0 (3)	C30—C29—H29A	113.6
C3—C11—H11A	113.6	C21—C29—H29B	113.6
C12—C11—H11A	113.6	C30—C29—H29B	113.6
C3—C11—H11B	113.6	H29A—C29—H29B	110.9
C12—C11—H11B	113.6	C32—C30—C31	111.0 (3)
H11A—C11—H11B	110.9	C32—C30—C29	116.2 (3)
C14—C12—C13	110.4 (3)	C31—C30—C29	111.6 (3)
C14—C12—C11	111.6 (3)	C32—C30—C22	115.4 (3)
C13—C12—C11	116.6 (3)	C31—C30—C22	113.7 (3)
C14—C12—C4	114.5 (3)	C29—C30—C22	87.0 (3)
C13—C12—C4	115.0 (3)	C30—C31—H31A	109.5
C11—C12—C4	87.2 (3)	C30—C31—H31B	109.5
C12—C13—H13A	109.5	H31A—C31—H31B	109.5
C12—C13—H13B	109.5	C30—C31—H31C	109.5
H13A—C13—H13B	109.5	H31A—C31—H31C	109.5
C12—C13—H13C	109.5	H31B—C31—H31C	109.5
H13A—C13—H13C	109.5	C30—C32—H32A	109.5
H13B—C13—H13C	109.5	C30—C32—H32B	109.5
C12—C14—H14A	109.5	H32A—C32—H32B	109.5
C12—C14—H14B	109.5	C30—C32—H32C	109.5
H14A—C14—H14B	109.5	H32A—C32—H32C	109.5
C12—C14—H14C	109.5	H32B—C32—H32C	109.5
H14A—C14—H14C	109.5	O6—C33—O5	121.9 (3)
H14B—C14—H14C	109.5	O6—C33—C34	125.6 (3)
O2—C15—O1	122.5 (3)	O5—C33—C34	112.5 (3)
O2—C15—C16	126.8 (3)	C33—C34—H34A	109.5
O1—C15—C16	110.8 (3)	C33—C34—H34B	109.5
C15—C16—H16A	109.5	H34A—C34—H34B	109.5
C15—C16—H16B	109.5	C33—C34—H34C	109.5
H16A—C16—H16B	109.5	H34A—C34—H34C	109.5
C15—C16—H16C	109.5	H34B—C34—H34C	109.5
H16A—C16—H16C	109.5	C25—C35—H35A	109.5
H16B—C16—H16C	109.5	C25—C35—H35B	109.5
C7—C17—H17A	109.5	H35A—C35—H35B	109.5
C7—C17—H17B	109.5	C25—C35—H35C	109.5
H17A—C17—H17B	109.5	H35A—C35—H35C	109.5
C7—C17—H17C	109.5	H35B—C35—H35C	109.5
H17A—C17—H17C	109.5	O8—C36—H36A	109.5
H17B—C17—H17C	109.5	O8—C36—H36B	109.5
O4—C18—H18A	109.5	H36A—C36—H36B	109.5
O4—C18—H18B	109.5	O8—C36—H36C	109.5
H18A—C18—H18B	109.5	H36A—C36—H36C	109.5
O4—C18—H18C	109.5	H36B—C36—H36C	109.5
			
C1—C2—C3—C11	30.8 (5)	C19—C20—C21—C29	16.6 (5)
C10—C2—C3—C11	−149.5 (3)	C28—C20—C21—C29	−162.5 (3)
C1—C2—C3—C4	136.7 (4)	C19—C20—C21—C22	123.1 (4)
C10—C2—C3—C4	−43.7 (4)	C28—C20—C21—C22	−56.0 (4)
C2—C3—C4—C5	95.8 (4)	C20—C21—C22—C23	94.6 (4)
C11—C3—C4—C5	−141.4 (3)	C29—C21—C22—C23	−141.2 (3)
C2—C3—C4—C12	−140.7 (3)	C20—C21—C22—C30	−142.7 (3)
C11—C3—C4—C12	−17.9 (3)	C29—C21—C22—C30	−18.5 (3)
C15—O1—C5—C4	−172.9 (3)	C33—O5—C23—C22	−172.0 (3)
C15—O1—C5—C6	64.7 (3)	C33—O5—C23—C24	67.2 (3)
C3—C4—C5—O1	162.8 (3)	C21—C22—C23—O5	164.6 (3)
C12—C4—C5—O1	54.8 (3)	C30—C22—C23—O5	57.8 (3)
C3—C4—C5—C6	−78.9 (4)	C21—C22—C23—C24	−77.9 (4)
C12—C4—C5—C6	173.1 (3)	C30—C22—C23—C24	175.3 (3)
O1—C5—C6—O3	37.1 (4)	O5—C23—C24—O7	41.2 (4)
C4—C5—C6—O3	−78.1 (4)	C22—C23—C24—O7	−75.2 (4)
O1—C5—C6—C7	−140.5 (3)	O5—C23—C24—C25	−134.8 (3)
C4—C5—C6—C7	104.3 (3)	C22—C23—C24—C25	108.8 (3)
O3—C6—C7—C17	−2.6 (5)	O7—C24—C25—C35	6.2 (5)
C5—C6—C7—C17	174.9 (3)	C23—C24—C25—C35	−177.9 (3)
O3—C6—C7—C8	127.1 (4)	O7—C24—C25—C26	136.3 (3)
C5—C6—C7—C8	−55.3 (4)	C23—C24—C25—C26	−47.9 (4)
C18—O4—C8—C9	164.3 (3)	C36—O8—C26—C27	167.2 (3)
C18—O4—C8—C7	−71.8 (4)	C36—O8—C26—C25	−69.1 (4)
C6—C7—C8—O4	172.0 (3)	C24—C25—C26—O8	165.2 (3)
C17—C7—C8—O4	−59.2 (4)	C35—C25—C26—O8	−65.3 (4)
C6—C7—C8—C9	−70.9 (4)	C24—C25—C26—C27	−77.6 (4)
C17—C7—C8—C9	57.9 (4)	C35—C25—C26—C27	51.9 (4)
O4—C8—C9—C10	−171.6 (3)	O8—C26—C27—C28	176.2 (3)
C7—C8—C9—C10	67.6 (4)	C25—C26—C27—C28	55.8 (4)
C1—C2—C10—C9	104.1 (4)	C19—C20—C28—C27	115.6 (4)
C3—C2—C10—C9	−75.6 (4)	C21—C20—C28—C27	−65.4 (4)
C8—C9—C10—C2	68.8 (4)	C26—C27—C28—C20	79.2 (4)
C2—C3—C11—C12	141.2 (3)	C20—C21—C29—C30	142.0 (3)
C4—C3—C11—C12	18.1 (3)	C22—C21—C29—C30	18.7 (3)
C3—C11—C12—C14	97.3 (3)	C21—C29—C30—C32	−135.5 (3)
C3—C11—C12—C13	−134.6 (3)	C21—C29—C30—C31	95.8 (3)
C3—C11—C12—C4	−17.9 (2)	C21—C29—C30—C22	−18.5 (3)
C5—C4—C12—C14	31.1 (4)	C23—C22—C30—C32	−99.7 (4)
C3—C4—C12—C14	−94.8 (3)	C21—C22—C30—C32	136.1 (3)
C5—C4—C12—C13	−98.2 (4)	C23—C22—C30—C31	30.2 (4)
C3—C4—C12—C13	135.9 (3)	C21—C22—C30—C31	−94.0 (3)
C5—C4—C12—C11	143.7 (3)	C23—C22—C30—C29	142.5 (3)
C3—C4—C12—C11	17.8 (2)	C21—C22—C30—C29	18.3 (2)
C5—O1—C15—O2	−0.6 (5)	C23—O5—C33—O6	2.8 (5)
C5—O1—C15—C16	178.2 (3)	C23—O5—C33—C34	−177.0 (3)
